# Medial Thigh Contouring in Massive Weight Loss: A Liposuction-Assisted Medial Thigh Lift 

**DOI:** 10.29252/wjps.8.2.171

**Published:** 2019-05

**Authors:** Verdiana Di Pietro, Marcello Colicchia Gianfranco, Valerio Cervelli, Pietro Gentile

**Affiliations:** 1 Department of Plastic, Reconstructive and Regenerative Surgery, University of Rome “Tor Vergata”, Italy;; 2 Department of Plastic and Reconstructive Surgery, Catholic University, “Our Lady of Good Counsel”, Tirane, Albania

**Keywords:** Medial thigh contouring, Weight loss, Liposuction-Assisted Medial Thigh Lift

## Abstract

**BACKGROUND:**

Thigh’s lifting can be associated with significant complications, if the medial thigh excess is removed en bloc. In this study, the liposuction-assisted medial thigh’s lift (LAMeT) procedure, outcomes and complications were assessed.

**METHODS:**

Twenty four females between 25 and 61 years with grade 2 or 3 on Pittsburgh Rating Scale (PRS) treated with medial thigh’s reduction were enrolled. Medial thigh’s reduction was performed in three different procedures of vertical, horizontal and LAMeT. Vertical thigh’s lift with fascia suspension was conducted in 13 patients with grade 3 of ptosis on PRS; horizontal thigh’s lift with fascia suspension was undertaken in 3 patients with grade 2 on PRS; vertical and horizontal thigh’s lift considered as control group was described as excision-only group; and LAMeT was performed in 8 patients with grade 2 and 3 on PRS.

**RESULTS:**

Complications were observed in 62.5% of patients who underwent vertical or horizontal thigh’s lift with fascia suspension and in 16.7% who experienced the LAMeT without fascia suspension. The most frequent complication was seroma. Hospital stay was significantly lower in the LAMeT.

**CONCLUSION:**

Medial thigh’s lift is a safe and satisfying procedure because it provides aesthetic improvement in massive weight loss patients. The complication rate is higher when skin excess and laxity are removed en bloc, as the resection of excess tissue is poorly selective. The LAMeT preserves lymphatic and blood vessels and allows a more anatomical resection of the excess skin. Thus postoperative complications incidence is lower and the patient heals faster.

## INTRODUCTION

The growing popularity of bariatric surgery was determined by the increase in the massive weight loss population. If substantial weight loss occurs, the skin laxity remaining at the level of arms, abdomen, hips and thighs can be significant enough to cause functional impairment, recurrent skin infections, as well as considerable psychological discomfort. Therefore, more people need plastic surgery to restore the shape and the harmony of the body. From a surgical point of view, the inner thigh is a problematic body region for various reasons. (i) It is a humid area, so wounds tend to heal more slowly; (ii) The skin is thin and weak; therefore, wound complications are likely to happen; (iii) The closeness of the anus and of the external genitals increases the risk of infection of the wound; (iv) The lymphatic vessels are very superficial and there is a high risk of lymphedema if are damaged; (v) The superficial venous system can be easily damaged during the resection causing upper limb edema; (vi) The scars often tend to enlarge due to the high tension of the sutures; and (vii) Scar retraction in the inguinal fold may cause labia majora and vaginal deformation.^1^^,^^2^

The medial thigh’s lift was described for the first time by Lewis in 1966, however the procedure did not succeed due to postoperative complications such as migration and enlargement of the scars, deformation of the vulva and relapse of ptosis.^1^ Pitanguy twenty years later, proposed to anchor the anterior thigh flap to the pubis or to the muscle fascia to prevent the recurrence of the ptosis.^2^ Lockwood suspended the dermis of the skin flap to the Colles fascia to prevent complications such as dehiscence of the wounds, scar enlargement and migration and ptosis recurrence.^3^ Since then other techniques were introduced in the attempt to correct the skin laxity, reduce the postoperative complications and have good aesthetic results.^4^^-^^8^

Le Louarn and Pascal described their own technique of medial thigh’s lift and used a horizontal scar located along the inguinal fold that never descended into the gluteal crease, reduced thigh volume with liposuction and removed the cutaneous excess with surgical resection of the skin.^9^ This technique introduced important innovations including liposuction that was performed on the entire thigh but aggressively under the area of resection to remove all the fat between the skin and the fascia; while, there was no dissection of tissues to be mobilized exclusively by liposuction.^9^ The direction of stretching the skin was concentric in order to reduce the length of the incision and distribute the tension throughout the horizontal scar; lastly, the positioning of anchoring sutures to the Colles fascia reducing the tension on the suture and the probability of scar complications.^9^

Despite its wide use and the evolution of the technique, the medial thigh’s lift still remains a procedure full of pitfalls especially in massive weight loss that presents further problems such as very thin and poor quality skin, excess skin extended to the knee or below, nutritional and vitamin deficiencies due to the bariatric intervention that predispose the patient to a slow and abnormal wound healing and bad quality scars.^10^ Sisti *et al*.^10^ reported a complication rate of 42.72%, among which the most frequent were the wound dehiscence (18.34%) and the seroma (8.05%).

In the last two years, the technique of medial thigh’s lift we has been evolved in massive weight loss, while at the beginning, Lockwood’s classic technique with horizontal or vertical scar and Colles fascia suspension was used, and today instead, a vertical scar with no anchor suture and concomitant liposuction is applied. In this technique described as liposuction-assisted medial thigh lift (LAMeT), as the resection is predominantly vertical and the tension of the suture is redistributed over the whole medial scar, stress on the inguinal scar is minimal and there’s no need of Colles fascia suspension. Furthermore liposuction lightens thigh flap of the thighs, mobilizes the tissues without need of undermining and preserves vascular and lymphatic network of the medial region of the thighs.^10^ In this study, the results of LAMeT evaluated for the morbidity, complication rate and final aesthetic outcome were compared with the standard technique.

## MATERIALS AND METHODS

In this retrospective study from January 2011 to September 2017, a total of 24 female patients (age range of 25-61 years) were randomly selected, underwent medial thigh’s reduction after massive weight loss. Vertical thigh’s lift with fascia suspension, as traditional procedure was performed in 13 patients with grade 3 of ptosis on Pittsburg Rating Scale (PRS) and considered as control group. Horizontal thigh’s lift with fascia suspension, as traditional procedure, was undertaken in 3 patients with grade 2 on PRS (control group). Vertical and Horizontal thigh’s lift considered as control group was described as excision-only group. LAMeT without fascia suspension was conducted in 8 patients (study group) with grade 2 and 3 on PRS. Evaluated parameters including age, body mass index (BMI), method of weight loss, and complications rate were shown in Table 1. All procedures performed involving human participants were in accordance with the ethical standards of the institutional and national research committee and with the 1964 Helsinki declaration and its later amendments or comparable ethical standards. All patients provided written informed consent before participating in the study.

**Table 1:** Demographic data of the two groups

**Variable**	**Overall**	**Excision-Only**	**Liposuction-Assisted**	***p*** ** value** *
No. of patients	24	16	8	
Age at surgery, yr	44.16 (min: 25, max: 61)	44.44 (min: 25, max: 61)	43.63 (min: 36, max: 52)	0.83
Mean weight loss, kg	61.25 (min: 22, max: 91)	58.6 (min: 22, max: 91)	66.5 (min: 41, max: 90)	0.27
BMI at surgery, kg/m^2^	28 (min: 22, max: 34.16)	28 (min: 22, max: 34.16)	27.94 (min: 22.8, max: 33.29)	0.95
Maximum BMI, kg/m^2^	50.73 (min: 41.77, max: 63.29)	49.45 (min: 41.77 max: 63.29)	53.3 (min: 47, max: 60.95)	0.15
Change in BMI, kg/m^2^	23.88 (min: 14.77, max: 36.45)	23.51 (min: 14.77, max: 36.45)	24.61 (min: 16.35, max: 32.27)	0.69
No. of comorbidities	14	11	3	

BMI: Body mass index;

* Statistically significant difference, *p*<0.05

The level of evidence was III as evidences were obtained from well-designed cohort or case-control analytic studies. Eligibility criteria were cutaneous ptosis after massive weight loss and when the patient’s skin was elastic enough for the resection to be done. Patients with massive weight loss were defined as those who had lost at least 22.67 kg of weight and maintained a stable weight for 3 months. The exclusion criteria were divided into two types of local and general. General exclusion criteria were blood, platelet, pro-thrombotic, and vascular disorders, bone marrow aplasia, uncompensated diabetes, sepsis and cancer. Local exclusion criteria were infection, cancer, loss of substance, deep vein thrombosis, and lymphoedema. Patients were excluded if the skin was not elastic enough, when they were obese. Fourteen patients had comorbidities (3 were in the LAMeT group, 11 were in the control group), while 16 patients were smokers (6 patients in the LAMeT group, and 11 patients in the control group). The average follow-up was 44 months (range: 4–120 months).

Regarding the tissue deformity and skin laxity on PRS, Song *et al*. have reported in 2005 an illustrative classification system that helps in systematical assessment and quantifying the level of tissue deformities.^11^ Ten anatomical regions were delineated for analysis including abdomen, arms, breast, flank, back, buttocks, hips, medial, lateral and lower thighs, and knees. A four-point grading scale was designed for each region. Grade 0 as normal range; grade 1 for mild deformity; grade 2 considered moderate deformity and grade 3 was regarded severe deformity. Generally, a mild deformity would require non-excisional or a minimally invasive procedure; a moderate one would need an excisional procedure; while a severe deformity would need combinations of excision and lifting, and would involve large areas of undermining.

Superficial lymphatic structures and the great saphenous vein and its branches underwent special attention, while performing a medial thigh lift. The lymphatics of the leg are primarily concentrated medially and lie deeper than the saphenous vein until they coalesce in the femoral triangle. An injury to the lymphatics can lead to disabling lower limb edema, which is usually permanent. In patients with significant varicose vein problems, the saphenous vein may also be excised along with medial skin resection.^11^

A standard technique according to Lockwood was established. Pre-operative markings were made with the patient standing in an upright position. The upper incision line was placed at the level of the inguinal fold approximately 1 centimetre (cm) caudally to avoid any distortion of the labia majora. The lateral apex of the ellipse reached about half of the thigh, while the medial one went up to the gluteal fold, without ever crossing it. The ellipse thickness was calculated by pinch test. In patients with grade 2 of ptosis, the horizontal scar was used. In case of significant ptosis (grade 3), a vertical scar was added. In this case, pinch test determined the quantity of skin to be removed and an ellipse was drawn, along the medial surface of the thigh starting from the perineal fold. Then a vertical line was drawn at the center of this ellipse, which roughly corresponded to the final scar. In some patients it was necessary to extend the incision up to the knee, below the medial condyle.

The procedure began with the incision of the proximal line; then a superficial dissection was performed to preserve the subcutaneous lymphatic network in the femoral triangle. After the resection of the horizontal ellipse and, when present, of the vertical one, the dermo-adipose flap of the thigh was anchored with permanent sutures to the Colles fascia to reduce the tension on the scar and obtain longer lasting results over time. A drain was left for each thigh treated and then, the planned suture of the subcutaneous tissue, subdermal tissue, and skin with absorbable suture Monocryl® 2-0, 3-0 and 4-0 were completed and later a compressive dressing was applied. Drains were removed when the amount of fluids collected was lower than 50 mL in 24 hours. 

**Fig. 1:**
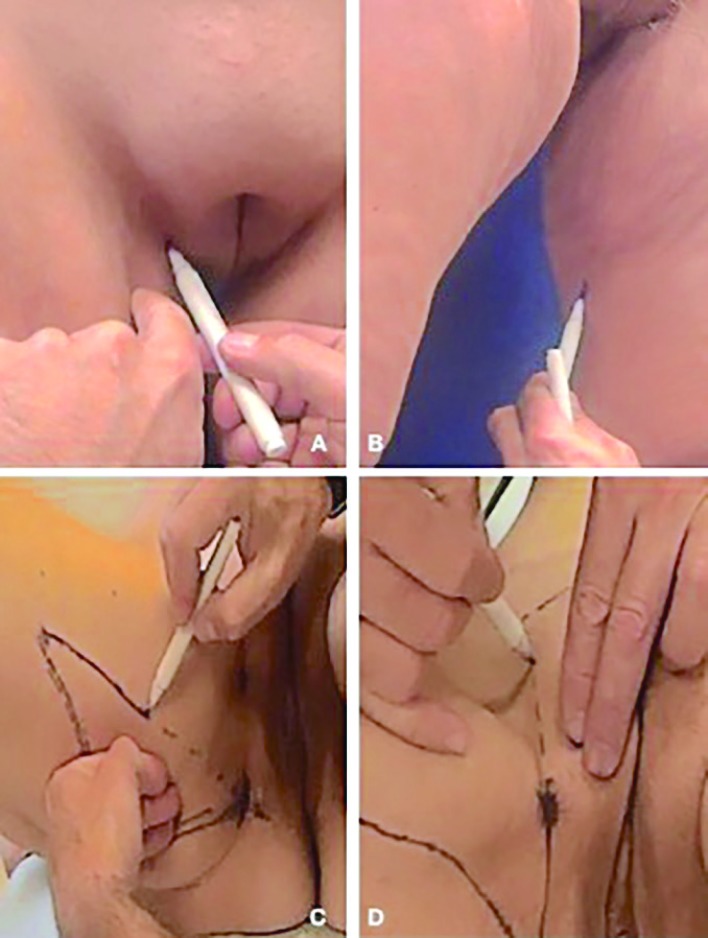
LAMeT preoperative markings. **(A)** Identification of the adductorcord. **(B)** Position of the ideal scar. **(C)** The ellipse of vertical resection. **(D)** Markings in the pubis area

Regarding LAMeT, the patient was drawn in a vertical position with legs abducted. The line corresponding to the final scar was marked, while the proximal point of this line was about 1 cm inside the adductor cord (Figure 1A). The distal point was instead placed along the medial surface of the leg and corresponded to the end of the skin fold. The two points were joined to identify the ideal position of the scar so that it would be well hidden inside the thighs (Figure 1B). As the patient was lying down, a horizontal line was drawn along the inguinal fold and up to the adductor cord taking care to draw the line 1 cm outer the fold to prevent the scar from causing a distortion of the labia majora.

The back line of the resection ellipse was drawn by firmly pulling the excess of the skin forward and tracing a straight line from the upper to the lower point. The anterior line was then drawn by firmly pulling the excess skin backward and tracing a straight line from the upper to the lower point (Figure 1C). After that, a point was marked and placed at 7 cm from the vulva and other two points were placed 7 centimetres laterally to the previous one (Figure 1D) and this point was joined to the adductor cord. Sometimes, the line was drawn in a more oblique direction to avoid too much vertical scars on the pubis. 

The extent of horizontal resection was not calculated preoperatively, but was dependent on the excess skin produced at the inguinal level by the vertical resection. The patient wore antithrombotic stockings or elastic wraps throughout the duration of the surgery as a preventive measure from thromboembolic risk. All patients received general anesthesia and were positioned supine. Leg supports were not used, so the limbs could be mobilized during surgery. Two grams of cefazolin was infused intravenously before the incision. Great care was taken to ensure that the patient’s body temperature remained adequate throughout the surgery avoiding a too low temperature in the operating room, covering the patient with blankets, using a heated bed and heating the infusion fluids.

**Fig. 2:**
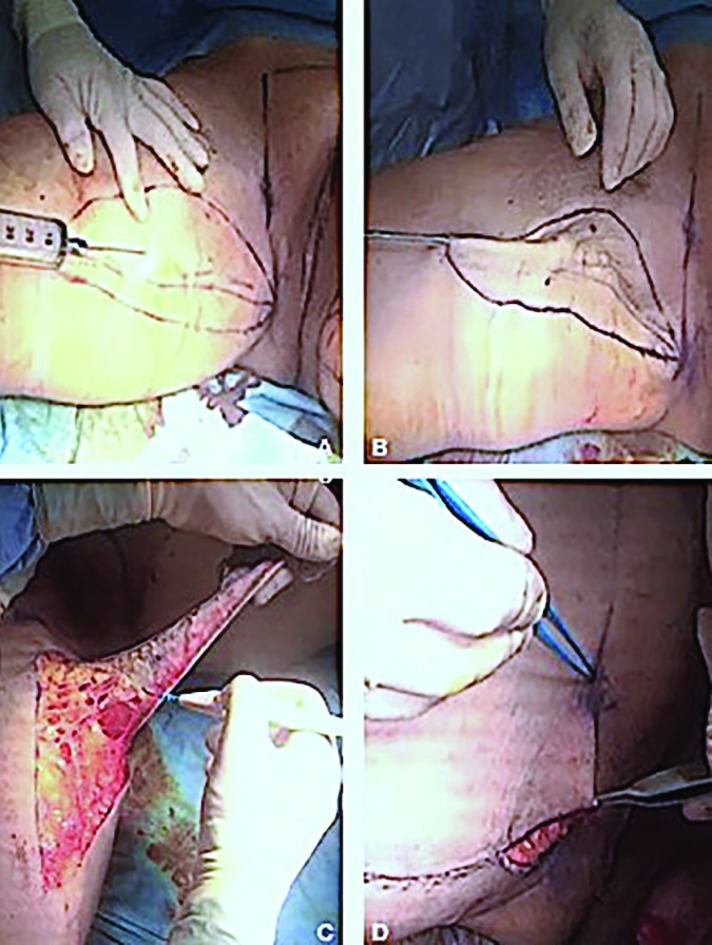
LAMeT surgical technique step by step. **(A)** Liposuction area injection. **(B)** Liposuction of the ellipse of resection: all the subcutaneous fat has been removed. **(C)** Skin resection. **(D)** Resection of the excess skin in the pubis area

The liposuction area (Figure 2A) was infiltrated with 1 mg of epinephrine/L saline solution. The infiltration–aspiration ratio was 1:1. Then the incision lines were infiltrated with 1.5 mg epinephrine/L saline solution. After a period of 15-20 minutes from the infiltration, the liposuction was performed in the area of skin resection, using three-hole and 4, 5, 6 mm diameter cannulas. All the fat tissues were removed between the skin and the muscle fascia going about 1 cm beyond the incision lines to facilitate closure. At the end of liposuction, the skin must be 2 or 3 millimetres (mm) thick (Figure 2B). If necessary, a “standard” liposuction (final thickness of the skin of 1-1.5 cm) was performed in the other parts of the thigh.

After liposuction, some silk suture was put and checked if the ellipse was wide enough and if it was possible to be closed. In that case, the anterior line was modified because the skin of the anterior thigh was looser and because to prevent the scar from moving anteriorly and becoming more visible. When satisfied with the drawing, the ellipse was cut and the incision was bloodless. The ellipse was then removed at a very superficial level, just below the dermal layer. It was important to pay attention to the inclination of the coagulator that must be facing the skin (Figure 2C). 

In this way, to make sure not to damage any important anatomical structure and not to cut too deeply. Once the ellipse was removed, the closure was simulated with staples or external silk stitches to make the definitive closure easier. At the level of the upper third, it was important during the closure to lift the front edge about 2 cm upwards to better stretch the skin. This displacement of the flap produced redundant skin in the inguinal fold. The limits were marked of the horizontal resection, orienting them along the line drawn at the pubic level during the preoperative markings (Figure 2D). 

After infiltration and liposuction, the excess skin was removed, always maintaining a very superficial dissection plane, immediately below the skin layer. As evidenced by the description of the technique, the tissues were not undermined, but were mobilized by liposuction. The absence of dead spaces as well as the integrity of the lymphatic and vascular network due to liposuction allowed not to use drains. The surgery was completed with the suture of the ellipse and a continuous suture in Vicryl2/0 was used and a single layer in the dermis was undertaken, since the skin of the massive weight loss subjects was very thin. 

Making knots both at the beginning and at the end of the suture was avoided to minimize dermal blood impairments. The suture was blocked at one end with two passages of the thread at the same level and if possible through the thread itself; at the end, four passages of the thread were made in the opposite direction. It is important that the needle enters and exits the skin perpendicularly in order to leave within the dermis with the least amount of thread and minimize the inflammation produced by the suture. Furthermore, the needle exited at the dermo-epidermal junction in order to obtain optimal margin juxtaposition. After closing, a semi-compressive dressing and an elastic garment awere then applied.

For post-operative treatment, suction drains were used in the vertical procedure, while a Penrose drains was applied in the horizontal procedure. In all patients, broad-spectrum antibiotic therapy was administered and thrombosis prophylaxis was implemented with the subcutaneous administration of enoxaparin sodium (Low-molecular-weight heparin) for 10 days with leg wrapping and mobilization on the second postoperative day. A Dermatix gel was used in the case of a hypertrophic scar. The patients were recommended to wear the garments night and day for at least 3 weeks. The patient was checked every 5 days for the first 15 days to replace the medication and every week for the first month. Other controls were made at 3, 6, 12 months and then annually. The average follow-up was 44 months (range: 4-120 months). Statistical analysis was performed using Microsoft Excel and SPPS software (Version 16, Chicago, IL, USA). A descriptive analysis of all data was first carried out and then, a comparative study was conducted between the LAMeT group and excision-only control group. Categorical variables were compared with Chi-Square analysis or Fisher exact test. Differences between groups were considered significant if *p* was less than 0.05. 

## RESULTS

The patients’ mean age was 44.16 years, mean weight at the time of surgery was 81.3 kg, with an average BMI of 28 kg/m^2^ (range: 22-34,16 kg/m^2^), while the maximum BMI was 50.73 kg/m^2^ (range: 41.77-63.29 kg/m^2^) and delta-BMI was 23.88 kg/m^2^ (range: 14.77-36.45 kg/m^2^). The mean weight loss was 61.25 kg (average delta-BMI: 19.56 kg). In all patients, the weight loss was obtained from bariatric surgery reports (Table 1). The mean time of surgery was 4.3 h in both groups. The hospital stay (2 days versus 4 days, range: 2-7 days) and time to drain removal (2.18 days versus 0.25 days) was lower in the LAMeT group. The mean amount of tissue removed was lower in the LAMET group (385.63 g, range 100-2100 g versus 715.75 g, range: 180-1959 g) as a consequence of the liposuction; mean aspirated volume was 1587.5 mL (range: 800-2200 mL, Table 2). The minimum follow-up range was 3 months.

**Table 2:** Surgical data of the two groups

**Variable**	**Overall**	**Excision-only**	**Liposuction-aAssisted**	***p*** ** value** *
No. of patients	24	16	8	
Duration, min	238.33 (min: 60, max: 375)	242.19 (min: 60, max: 360)	230 (min: 190, max: 375)	0.73
Lipoaspirate, mL	1058.33 (min: 0, max: 2200)	0	1587.5 (min: 800, max: 2200)	9.36
Resection weight, g	605.71 (min: 100, max: 2100)	715.75 (min: 180, max: 1950)	385.62 (min: 100, max: 2100)	0.18
Time to drain removal, days	1.54 (min: 0, max: 5)	2.18 (min: 0, max: 5)	0.25 (min: 0, max: 2)	0.008
Days hospital stay, days	4 (min: 2, max: 7)	5 (min: 3, max: 7)	2	1.14

* Statistically significant difference, *p*<0.05

**Fig. 3:**
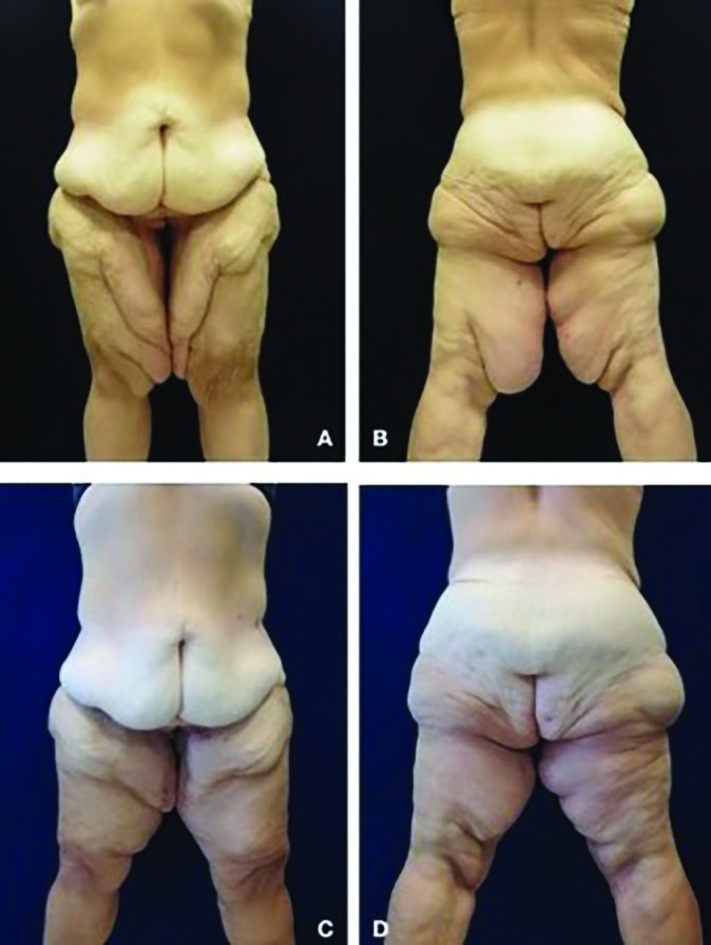
**(A-B)** Anterior and posteriorview of this 50-year-old woman who underwent LAMeT after gastric bypass and weight loss of 80 kg. **(C-D)** Three months postoperatively. Skin excess had been removed with functional and aesthetic improvement

**Fig. 4:**
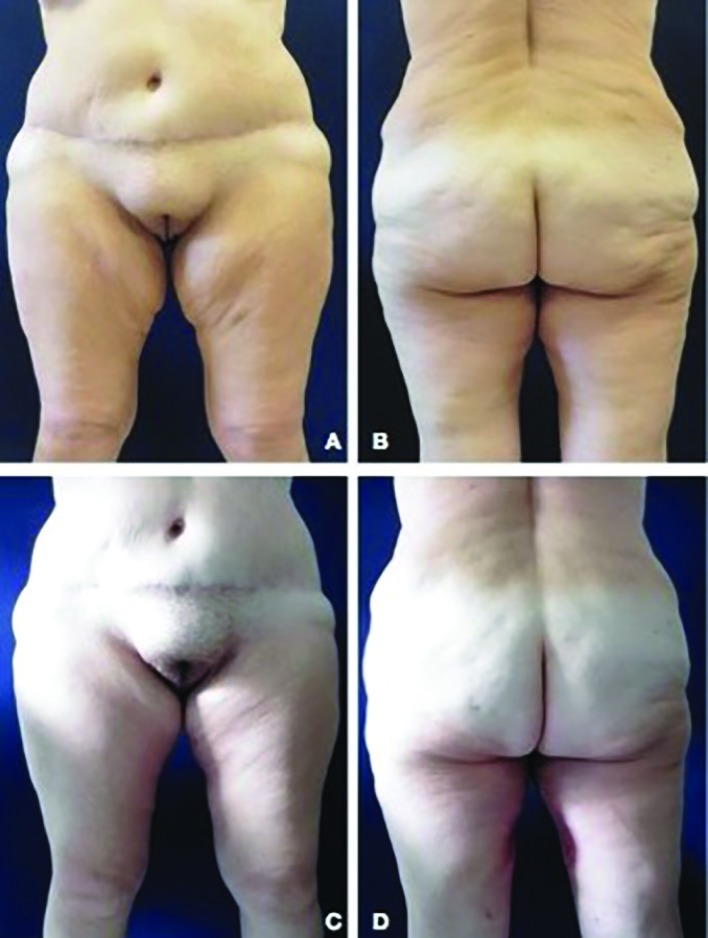
**(A-B)** Anterior and posteriorview of this 52-year-old woman who underwent Liposuction Assisted Medial Thigh Lift after gastric bypass and weight loss of 50 kg. **(C-D)** Three months postoperatively. Note the location and the quality of the scar

The incidence of postoperative complications was globally 45% (11 patients); the incidence of complications was significantly lower in the LAMeT group [1 patient (16.7%) versus 10 patients (62.5%); *p*<0.05] (Table 3). No major complications were observed in either group. The incidence of seroma was significantly lower in the LAMET group (0 patients versus 8), while no statistically significant findings were observed for hematoma, infection or dehiscence. Only one patient required a second surgical treatment and all the complications were managed on an outpatient basis. The seroma was treated by fine needle aspirations, while the cases of wound infection and dehiscence were treated with local medications and oral antibiotics. A total of 9 patients had problems of scar enlargements or migration, 8 in the excision-only group (control group) versus 1 in the LAMeT. None of patients experienced deformation of the vulva. Risk factors for minor complications have found to be smoking (odds ratio 32, *p*<0.05) and standard technique (odds ratio: 11.67, *p*<0.05). Representative cases of LAMeT technique were shown in Figure 3 and 4.

**Table 3:** Complications of both groups

**Variable**	**Overall**	**Excision-only**	**Liposuction-assisted**	***p*** ** value** *
No. of patients	24	16	8	>0.05
Seroma	8	8	0	<0.05
Hematoma	2	2	0	>0.05
Wound dehiscence	9	9	0	<0.05
Wound infection	1	0	1	>0.05
Surgical revision	1	1	0	>0.05
Total	13	10	3	>0.05

* Statistically significant difference, *p*<0.05

## DISCUSSION

The thigh’s lifting is a surgical procedure used to treat post-bariatrics patients to remove the excess skin of the thighs following the massive weight loss. The entity of the deformity is classified according to the PRS, which identifies three degrees of deformities. The first degree is the ‘non-deflated type’ that has an excess of volume in the absence of skin laxity; the second degree is the ‘mixed type’ with a moderate excess of skin and volume; and finally, the third degree is the ‘deflated type’, with little excess volume, but considerable skin excess. In our study, only patients with grade 2 and 3 of the PRS were enrolled.^12^

In these patients, the high risk of postoperative complications has led in recent years to an innovative approach that combines liposuction and surgical excision. After the encouraging results of Saldanha^13^ and Avelar^14^ in lipo-abdominoplasty and the positive experience of Le Louarn and Pascal in medial lift thigh lift,^9^ in recent years other authors have proposed the use of liposuction combined with body contouring techniques.^15^^-^^17^ In fact, liposuction reduces the volume of the thighs without damaging the lymphatic, vascular and nervous structures and as consequence it may reduce the incidence of postoperative complications.^15^^-^^17^

Several studies have shown that liposuction preserves perforating vessels wider than 1 mm. Salgarello demonstrated how the perforating vessels of the deep inferior epigastric artery were equal in number, diameter and flow before and 6 months after liposuction.^18^ Similar results were reported by Graf *et al.* about the perforators of the abdominal skin.^19^ The fact that we have had no cases of skin necrosis or postoperative persistent edema clearly confirms the benefits of liposuction. In a recent study, Bertheuil *et al.* demonstrated how liposuction also preserved the microcirculation where gas and molecular exchanges occurred between blood and tissues.^20^ In this study, the authors showed that the concentration of endothelial cells was markedly greater in the adipose tissue excreted en bloc than the aspirated one. 

Furthermore, in the residual tissue after liposuction, the vessels up to 30 mm in diameter were intact and no larger vessels were found in the aspirated adipose tissue. These results proved that liposuction removed fat in a selective way preserving the micro-vascular and lymphatic structures that were instead cut together with the adipose tissue, when the excision was performed en bloc. A particularly fearsome complication of the medial thigh lift was the seroma that might require multiple drains or even surgery. In our study, the incidence of this complication was significantly lower in the LAMeT group because liposuction preserved the lymphatic and vascular structures and there was no tissue undermining. 

This avoids creation of dead spaces that the body tends to fill with fluids and also avoids injury of blood and lymphatic vessels.^21^^-^^23^ We did not observe any differences in duration of the intervention, because the liposuction was performed on a very limited surface. So it was completed by the surgeon in a short time; furthermore, the cut was bloodless, in this way, we proceeded faster as we should not coagulate the dermis. Another innovative aspect to consider was that in the LAMeT group, drains was not used leading to an increase in the patient comfort and at the same time reduced the risk of wound infection that in fact was very low. In addition, the lower morbidity of the procedure reduced the average length of hospital stay, allowed patients to return earlier to their work activities and reduced costs related to hospitalization.

A complication reported frequently in the literature in the medial thigh lift is the dehiscence of the sutures caused by the combination of several factors such as thin and poor quality skin, high local humidity, high frequency of infections and especially high tension of the sutures.^24^^,^^25^ In his initial description, Lewis proposed a technique with horizontal resection in which the traction vector was predominantly vertical, so that all the weight of the skin flap was supported by the inguinal suture; not by chance.^1^

There was a high frequency of dehiscence of the sutures, of migration of scars and deformation of the labia majora. To avoid these complications, a technique of fascial suspension was proposed a few years later, in which the raised flap was anchored to the Colles fascia.^25^ In the LAMeT, the authors have modified the traction vector that was no longer just vertical, but vertical and horizontal. This allowed to redistribute the tension over the entire length of the vertical scar and there was no need of Lockwood fascial suspension. The vertical scar also allowed better lifting of the distal third of the thigh and of the knee region which are not treated with the traditional technique. 

Some patients may refuse surgery because of the scar length and location. Therefore, it is necessary to explain that the scar is well hidden in frontal vision and it is necessary, if we want to obtain a better and longer lasting result. The lower incidence of wound dehiscence is also due to a lower thermal damage on the dermis. The cut was bloodless and there was no need to coagulate the dermis which then healed better and faster. The absence of thermal dermis injury also improved the quality of the scars. In the LAMeT group, no cases of scar enlargements or migration were observed. Other studies are needed to give definitive conclusions on LAMeT, however, positive results encourage the use of the technique especially in patients after massive weight loss.

The medial thigh’s lift is a really popular surgical procedure among post-bariatric patients because the cutaneous excess in the inner thigh can cause functional impairment, frequent cutaneous infections, as well as psychological distress. However, even if widespread, it is associated with a high incidence of postoperative complications. The LAMeT technique preserves the great majority of lymphatics and blood vessels and nerves and allows a more anatomical resection of the excess skin. Thus it reduces postoperative complications and allows the patient to heal faster. The encouraging results of the LAMeT as well as patient satisfaction have convinced the authors to completely abandon the standard technique.

## CONFLICT OF INTEREST

The authors declare no conflict of interest.
